# 

**Published:** 1906-10-20

**Authors:** 


Young mothers and nurses charged with the upbringing of children from babyhood will find

### The Care of Children

By ROBERT J. BLACKHAM, D.P.H. (Lond.)

an invaluable help and guide to the general management of the nursery.

It contains practical Hints on Food, Drink, Medicine, Clothing, Cleanliness, Rest, Exercise, Infectious Diseases, 'First Aid,' Nursery Cookery, &c., &c., expressed in clear and simple language.

***Price 1/6 net; 1/8 post free.***

OF ALL BOOKSELLERS,

OR OF THE PUBLISHERS,

**The Scientific Press, Limited,**

28/29 SOUTHAMPTON STREET,

STRAND, LONDON, W.C.

### 2/6 NURSING MANUALS

By Post, 2/9

**ELEMENTARY ANATOMY AND SURGERY FOR NURSES.**

By William McAdam Eccles, M.S.Lond., M.B., F.R.C.S., L.R.C.P. Profusely Illustrated.

**NURSING IN DISEASES OF THE THROAT, NOSE AND EAR.**

By P. Macleod Yearsley, F.R.C.S.Eng., M.R.C.S., L.R.C.P.Lond. Illustrated.

**LECTURES UPON THE NURSING OF INFECTIOUS DISEASES.**

By F. J. Woollacott, M.A., M.D.

**MEDICAL ELECTRICITY AND LIGHT TREATMENT.**

**A Practical Handbook for Nurses.**

By Kate Neale, Sister in Charge of the Actino-Therapeutic Department, Guy's Hospital. Profusely Illustrated.

**ELEMENTARY TREATISE ON THE LIGHT TREATMENT FOR NURSES.**

By James H. Sequeira, M.D.Lond. Illustrated.

**HOSPITAL SISTERS AND THEIR DUTIES.**

By Eva C. E. Lückes, Matron, London Hospital. Third Edition, enlarged and partly re-written.

**TENDENCIES TO CONSUMPTION:**

**How to Counteract Them.**

By J. D. E. Mortimer, M.B.Lond.; M.R.C.S., L.S.A., Author of "Home Nursing of Sick Children."

**SPINAL CURVATURE AND AWKWARD DEPORTMENT:**

**Their Causes and Prevention in Children.**

By Dr. George Müller, Professor of Medicine and Orthopædics, Berlin. English Edition, edited and adapted by Richard Greene, F.R.C.P.Ed. With Plates and Illustrations.

*Complete Catalogue post free on application.*

THE SCIENTIFIC PRESS, Ltd.,

Nursing Publishers and Booksellers,

28 & 29 SOUTHAMPTON STREET,

STRAND, LONDON, W.C.

